# Supplementary material for: Temozolomide Chronotherapy in Glioma: A Systematic Review
Source: Curr Oncol. 2023 Feb 4;30(2):1893–902. doi: 10.3390/curroncol30020147 (PMC9955138; doi:10.3390/curroncol30020147)
Supplement: Supplementary file 1 [file curroncol-30-00147-s001.zip › curroncol-2172282-supplementary-need update.pdf]

Table S1. Studies excluded after stage 1 and 2 screening with their reason for exclusion.

Reasons for exclusion key	
1	Does not meet eligibility criteria
2	Duplicate text
3	Not clinical study (review, guideline, protocol, letter)
4	Article inaccessible

Table S2. The details of article exclusion during second-stage screening.

Reference	Reason for Exclusion
Mohile, N. A., et al. (2022). "Therapy for Diffuse Astrocytic and Oligodendroglial Tumors in Adults: ASCO-SNO Guideline." Journal of clinical oncology : official journal of the American Society of Clinical Oncology 40(4): 403-426.	3
Peters, K. B., et al. (2022). "Effects of low-dose naltrexone on quality of life in high-grade glioma patients: a placebo-controlled, double-blind randomized trial." Supportive care in cancer : official journal of the Multinational Association of Supportive Care in Cancer 30(4): 3463-3471	1
Bautista, F., et al. (2021). "Phase I or II Study of Ribociclib in Combination With Topotecan-Temozolomide or Everolimus in Children With Advanced Malignancies: Arms A and B of the AcSe-ESMART Trial." Journal of clinical oncology : official journal of the American Society of Clinical Oncology 39(32): 3546-3560	1
Clement, P. M. J., et al. (2021). "Impact of depatuxizumab mafodotin on health-related quality of life and neurological functioning in the phase II EORTC 1410/INTELLANCE 2 trial for EGFR-amplified recurrent glioblastoma." European journal of cancer (Oxford, England : 1990) 147: 1-12	1
Guo, L., et al. (2021). "The efficacy of hypofractionated radiotherapy (HFRT) with concurrent and adjuvant temozolomide in newly diagnosed glioblastoma: A meta-analysis." Cancer radiotherapie : journal de la Societe francaise de radiotherapie oncologique 25(2): 182-190	3
Moloudizargari, M., et al. (2021). "Therapeutic targets of cancer drugs: Modulation by melatonin." Life Sciences 267: 118934	3
Schloss, J., et al. (2021). "A Phase 2 Randomised Clinical Trial Assessing the Tolerability of Two Different Ratios of Medicinal Cannabis in Patients With High Grade Gliomas." Frontiers in Oncology 11: 649555	1
Bouchart, C., et al. (2020). "Prognostic impact of glioblastoma stem cell markers OLIG2 and CCND2." Cancer medicine 9(3): 1069-1078	1
Climans, S. A., et al. (2020). "Prolonged response to vismodegib in a patient with systemic medulloblastoma metastases." BMJ case reports 13(10)	1
Jiang, H., et al. (2020). "Short-term outcomes and predictors of post-surgical seizures in patients with supratentorial low-grade gliomas." Journal of clinical neuroscience : official journal of the Neurosurgical Society of Australasia 72: 163-168	1
Kaley, T. J., et al. (2020). "Phase I clinical trial of temsirolimus and perifosine for recurrent glioblastoma." Annals of Clinical and Translational Neurology 7(4): 429-436	1
Kessler, T., et al. (2020). "Methylome analyses of three glioblastoma cohorts reveal chemotherapy sensitivity markers within DDR genes." Cancer medicine 9(22): 8373-8385	1
Poon, M. T. C., et al. (2020). "Longer-term (>= 2 years) survival in patients with glioblastoma in population-based studies pre- and post-2005: a systematic review and meta-analysis." Scientific reports 10(1): 11622	3
Richard, S., et al. (2020). "Dual MGMT inactivation by promoter hypermethylation and loss of the long arm of chromosome 10 in glioblastoma." Cancer medicine 9(17): 6344-6353	1
von Rosenstiel, C., et al. (2020). "Correlation of the quantitative level of MGMT promoter methylation and overall survival in primary diagnosed glioblastomas using the quantitative MethyQESD method." Journal of clinical pathology 73(2): 112-115	1

Yang, K., et al. (2020). "Clinical Features and Outcomes of Primary Spinal Cord Glioblastoma: A Single-Center Experience and Literature Review." World neurosurgery 143: e157-e165	3
Yumimoto, K. and K. I. Nakayama (2020). "Recent insight into the role of FBXW7 as a tumor suppressor." Seminars in Cancer Biology 67: 1-15	3
Adam, D. (2019). "Emerging science of chronotherapy offers big opportunities to optimize drug delivery." Proceedings of the National Academy of Sciences of the United States of America 116(44): 21957-51959.	3
Bani-Sadr, A., et al. (2019). "Combined analysis of MGMT methylation and dynamic-susceptibility-contrast MRI for the distinction between early and pseudo-progression in glioblastoma patients." Revue neurologique 175(9): 534-543	3
Norden, A. D., et al. (2019). "A Real-World Claims Analysis of Costs and Patterns of Care in Treated Patients with Glioblastoma Multiforme in the United States." Journal of managed care & specialty pharmacy 25(4): 428-436	3
Petrova, L., et al. (2019). "Cerebral blood volume and apparent diffusion coefficient - Valuable predictors of non-response to bevacizumab treatment in patients with recurrent glioblastoma." Journal of the Neurological Sciences 405: 116433	3
Santos-Pinheiro, F., et al. (2019). "Treatment and long-term outcomes in pituitary carcinoma: a cohort study." European journal of endocrinology 181(4): 397-407	1
Stazi, G., et al. (2019). "Dissecting the role of novel EZH2 inhibitors in primary glioblastoma cell cultures: effects on proliferation, epithelial-mesenchymal transition, migration, and on the pro-inflammatory phenotype." Clinical epigenetics 11(1): 173	3
Symonds, P. and G. D. D. Jones (2019). "Hot Topics in Radiobiology." Clinical Oncology 31(5): 269-271.	3
Wang, J., et al. (2019). "Radiotherapy versus radiotherapy combined with temozolomide in high-risk low-grade gliomas after surgery: study protocol for a randomized controlled clinical trial." Trials 20(1): 641	3
Actrn (2018). "Phase III Trial of Extended Temozolomide in Newly Diagnosed Glioblastoma." To determine if extended post-radiation temozolomide will improve survival outcomes in patients with newly diagnosed glioblastoma	3
Azizi, A. A., et al. (2018). "Does the interval from tumour surgery to radiotherapy influence survival in paediatric high grade glioma?" Beeinflusst die Zeitspanne zwischen Tumorresektion und Strahlentherapie das Uberleben bei Kindern mit hochgradigen Gliomen? 194(6): 552-559	1
Belin, C., et al. (2018). "Interest of QUID-7 for insular localization gliomas." Neuro-Oncology 20(Supplement 3)	1
Binabaj, M. M., et al. (2018). "The prognostic value of MGMT promoter methylation in glioblastoma: A meta-analysis of clinical trials." Journal of cellular physiology 233(1): 378-386	3
Iwashita, Y., et al. (2018). "Possibility of venoarterial extracorporeal membranous oxygenator being a bridging therapy for hemodynamic deterioration of pulmonary tumor thrombotic microangiopathy prior to initiating chemotherapy: A case report." Medicine 97(37): e12169	1
Izquierdo, C., et al. (2018). "Long-term impact of temozolomide on 1p/19q-codeleted low-grade glioma growth kinetics." Journal of neuro-oncology 136(3): 533-539	3
Kajitani, T., et al. (2018). "Three case reports of radiation-induced glioblastoma after complete remission of acute lymphoblastic leukemia." Brain tumor pathology 35(2): 114-122	1
Li, Y., et al. (2018). "miR-1268a regulates ABCC1 expression to mediate temozolomide resistance in glioblastoma." Journal of neuro-oncology 138(3): 499-508	1
Mrowczynski, O. D., et al. (2018). "Utility of Early Postoperative Magnetic Resonance Imaging After Glioblastoma Resection: Implications on Patient Survival." World neurosurgery 120: e1171-e1174	3
Murphy, E. S., et al. (2018). "Risk Factors for Malignant Transformation of Low-Grade Glioma." International journal of radiation oncology, biology, physics 100(4): 965-971	1
Nct (2018). "Phase II Trial of Seizure Prophylaxis in Suspected Primary Glioma Patients Undergoing Craniotomy." Phase II Trial of Seizure Prophylaxis in Suspected Primary Glioma Patients Undergoing Craniotomy	3

Nct (2018). "Safety and Efficacy Study of Trans Sodium Crocetinate (TSC) in Newly Diagnosed Glioblastoma (GBM) Biopsy-Only Subjects." Open-label, Randomized, Controlled, Phase 3 Safety and Efficacy Study of Trans Sodium Crocetinate With Radiation Therapy and Temozolomide in Newly Diagnosed Glioblastoma (GBM) Biopsy-Only Subjects	3
Ogawa, Y., et al. (2018). "Mid-term prognosis of non-functioning pituitary adenomas with high proliferative potential: really an aggressive variant?" Journal of neuro-oncology 137(3): 543-549	1
Peeples, L. (2018). "TIME TRIALS." Nature 556(7701): 290-292.Synchronizing drug delivery with a patient's body clock can yield clear benefits. But will the data be enough to overcome long-standing hurdles	3
Rapp, M., et al. (2018). "A randomized controlled phase II trial of vaccination with lysate-loaded, mature dendritic cells integrated into standard radiochemotherapy of newly diagnosed glioblastoma (GlioVax): study protocol for a randomized controlled trial." Trials 19(1): 293	3
Ronellenfitsch, M. W., et al. (2018). "Akt and mTORC1 signaling as predictive biomarkers for the EGFR antibody nimotuzumab in glioblastoma." Acta neuropathologica communications 6(1): 81	1
Shi, W., et al. (2018). "Investigating the Effect of Reirradiation or Systemic Therapy in Patients With Glioblastoma After Tumor Progression: A Secondary Analysis of NRG Oncology/Radiation Therapy Oncology Group Trial 0525." International journal of radiation oncology, biology, physics 100(1): 38-44	1
Sulli, G., et al. (2018). "Pharmacological activation of REV-ERBs is lethal in cancer and oncogene-induced senescence." Nature 553(7688): 351-355	1
van den Bent, M. J., et al. (2018). "Bevacizumab and temozolomide in patients with first recurrence of WHO grade II and III glioma, without 1p/19q co-deletion (TAVAREC): a randomised controlled phase 2 EORTC trial." The Lancet. Oncology 19(9): 1170-1179	1
Waschke, A., et al. (2018). "Cost-effectiveness of the long-term use of temozolomide for treating newly diagnosed glioblastoma in Germany." Journal of neuro-oncology 138(2): 359-367	3
Yin, J. Q., et al. (2018). "A case of temozolomide chemoradiotherapy induced pneumocystis jirovecii pneumonia in a non-HIV patient." American Journal of Respiratory and Critical Care Medicine 197	1
Badruddoja, M. A., et al. (2017). "Phase II study of bi-weekly temozolomide plus bevacizumab for adult patients with recurrent glioblastoma." Cancer chemotherapy and pharmacology 80(4): 715-721	1
Boxerman, J. L., et al. (2017). "Longitudinal DSC-MRI for Distinguishing Tumor Recurrence From Pseudoprogression in Patients With a High-grade Glioma." American journal of clinical oncology 40(3): 228-234	1
Conen, K. L., et al. (2017). "Prospective evaluation of stress in patients with newly diagnosed glioblastoma and in a close partner (TOGETHER-study)." Journal of Clinical Oncology 35(15 Supplement	1
Harris, G., et al. (2017). "Survival Outcomes of Elderly Patients With Glioblastoma Multiforme in Their 75th Year or Older Treated With Adjuvant Therapy." International journal of radiation oncology, biology, physics 98(4): 802-810	1
Liu, H.-P., et al. (2017). "Efficacy and safety of temozolomide plus whole-brain radiotherapy in the treatment of intracranial metastases." Journal of cancer research and therapeutics 13(5): 785-789	1
Marampon, F., et al. (2017). "HDAC4 and HDAC6 sustain DNA double strand break repair and stem-like phenotype by promoting radioresistance in glioblastoma cells." Cancer letters 397: 1-11	1
Nathan, J. K., et al. (2017). "Early initiation of chemoradiation following index craniotomy is associated with decreased survival in high-grade glioma." Journal of neuro-oncology 135(2): 325-333	1
Raj, N., et al. (2017). "O6-Methylguanine DNA Methyltransferase Status Does Not Predict Response or Resistance to Alkylating Agents in Well-Differentiated Pancreatic Neuroendocrine Tumors." Pancreas 46(6): 758-763	1
Shenouda, G., et al. (2017). "A Phase 2 Trial of Neoadjuvant Temozolomide Followed by Hypofractionated Accelerated Radiation Therapy With Concurrent and Adjuvant Temozolomide for Patients With Glioblastoma." International journal of radiation oncology, biology, physics 97(3): 487-494	1
Shiraki, Y., et al. (2017). "Significance of perivascular tumour cells defined by CD109 expression in progression of glioma." The Journal of pathology 243(4): 468-480	1

Slat, E. A., et al. (2017). "Cell-intrinsic, Bmal1-dependent Circadian Regulation of Temozolomide Sensitivity in Glioblastoma." Journal of biological rhythms 32(2): 121-129	1
Weller, M., et al. (2017). "Rindopepimut with temozolomide for patients with newly diagnosed, EGFRvIII-expressing glioblastoma (ACT IV): a randomised, double-blind, international phase 3 trial." The Lancet. Oncology 18(10): 1373-1385	1
Xue, W., et al. (2017). "Aberrant glioblastoma neovascularization patterns and their correlation with DCE-MRI-derived parameters following temozolomide and bevacizumab treatment." Scientific reports 7(1): 13894	1
Amiri, A., et al. (2016). "Inhibition of carbonic anhydrase IX in glioblastoma multiforme." European journal of pharmaceutics and biopharmaceutics : official journal of Arbeitsgemeinschaft fur Pharmazeutische Verfahrenstechnik e.V 109: 81-92	1
Ananta, J. S., et al. (2016). "Temozolomide-loaded PLGA nanoparticles to treat glioblastoma cells: a biophysical and cell culture evaluation." Neurological research 38(1): 51-59	1
Chinot, O. L., et al. (2016). "Upfront bevacizumab may extend survival for glioblastoma patients who do not receive second-line therapy: an exploratory analysis of AVAglio." Neuro-Oncology 18(9): 1313-1318	1
Dai, C., et al. (2016). "Refractory pituitary adenoma: a novel classification for pituitary tumors." Oncotarget 7(50): 83657-83668	3
Duma, C. M., et al. (2016). "Upfront boost Gamma Knife "leading-edge" radiosurgery to FLAIR MRI-defined tumor migration pathways in 174 patients with glioblastoma multiforme: a 15-year assessment of a novel therapy." Journal of Neurosurgery 125(Suppl 1): 40-49	1
Jhaveri, N., et al. (2016). "A novel drug conjugate, NEO212, targeting proneural and mesenchymal subtypes of patient-derived glioma cancer stem cells." Cancer letters 371(2): 240-250	1
Kaul, D., et al. (2016). "Accelerated hyperfractionation plus temozolomide in glioblastoma." Radiation oncology (London, England) 11: 70	1
Mizumoto, M., et al. (2016). "Proton beam therapy with concurrent chemotherapy for glioblastoma multiforme: comparison of nimustine hydrochloride and temozolomide." Journal of neuro-oncology 130(1): 165-170	1
Ros Bernaola, G., et al. (2016). "Chromatopsia and night blindness in a patient on capecitabine and temozolomide." European Journal of Hospital Pharmacy 23(Supplement 1): A148	1
Sardi, I. (2016). "Prolonged exposure to doxorubicin after radiotherapy in glioblastoma." Future oncology (London, England) 12(5): 581-583.	1
Schliesser, M. G., et al. (2016). "Prognostic relevance of miRNA-155 methylation in anaplastic glioma." Oncotarget 7(50): 82028-82045	1
Tian, T., et al. (2016). "MicroRNA-101 reverses temozolomide resistance by inhibition of GSK3beta in glioblastoma." Oncotarget 7(48): 79584-79595	1
Wang, Z., et al. (2016). "HDAC6 promotes cell proliferation and confers resistance to temozolomide in glioblastoma." Cancer letters 379(1): 134-142	1
Yang, C. H., et al. (2016). "MiRNA203 suppresses the expression of protumorigenic STAT1 in glioblastoma to inhibit tumorigenesis." Oncotarget 7(51): 84017-84029	1
Yang, S. H., et al. (2016). "Metformin treatment reduces temozolomide resistance of glioblastoma cells." Oncotarget 7(48): 78787-78803	1
Banelli, B., et al. (2015). "The histone demethylase KDM5A is a key factor for the resistance to temozolomide in glioblastoma." Cell cycle (Georgetown, Tex.) 14(21): 3418-3429	1
Bhattacharyya, G. S., et al. (2015). "Phase ii trial of metronomic temozolomide and topotecan in patients with relapsed and/or refractory small cell lung cancer." Journal of Thoracic Oncology 10(9 SUPPL. 2): S743	1
Boccard, S. G., et al. (2015). "Inhibition of DNA-repair genes Ercc1 and Mgmt enhances temozolomide efficacy in gliomas treatment: a pre-clinical study." Oncotarget 6(30): 29456-29468	1
Brodbelt, A., et al. (2015). "Glioblastoma in England: 2007-2011." European journal of cancer (Oxford, England : 1990) 51(4): 533-542	3

Brzozowska, A., et al. (2015). "The impact of surgery on the efficacy of adjuvant therapy in glioblastoma multiforme." Advances in clinical and experimental medicine : official organ Wroclaw Medical University 24(2): 279-287	1
David, C. N., et al. (2015). "Antitumor activity of a polypyridyl chelating ligand: in vitro and in vivo inhibition of glioma." ASN neuro 7(1)	1
Favero, F., et al. (2015). "Glioblastoma adaptation traced through decline of an IDH1 clonal driver and macro-evolution of a double-minute chromosome." Annals of oncology : official journal of the European Society for Medical Oncology 26(5): 880-887	1
Filippi-Chiela, E. C., et al. (2015). "Single-cell analysis challenges the connection between autophagy and senescence induced by DNA damage." Autophagy 11(7): 1099-1113	1
Fraser, E., et al. (2015). "New approaches in primary central nervous system lymphoma." Chinese clinical oncology 4(1): 11	3
Hassler, M. R., et al. (2015). "Thalidomide as palliative treatment in patients with advanced secondary glioblastoma." Oncology (Switzerland) 88(3): 173-179.	1
Kerschbaumer, J., et al. (2015). "Dual Anti-angiogenic Chemotherapy with Temozolomide and Celecoxib in Selected Patients with Malignant Glioma Not Eligible for Standard Treatment." Anticancer Research 35(9): 4955-4960.	1
Kessler, T., et al. (2015). "Glioma cell VEGFR-2 confers resistance to chemotherapeutic and antiangiogenic treatments in PTEN-deficient glioblastoma." Oncotarget 6(31): 31050-31068	1
Kovic, B. and F. Xie (2015). "Economic Evaluation of Bevacizumab for the First-Line Treatment of Newly Diagnosed Glioblastoma Multiforme." Journal of clinical oncology : official journal of the American Society of Clinical Oncology 33(20): 2296-2302	3
Krauze, A. V., et al. (2015). "A Phase 2 Study of Concurrent Radiation Therapy, Temozolomide, and the Histone Deacetylase Inhibitor Valproic Acid for Patients With Glioblastoma." International journal of radiation oncology, biology, physics 92(5): 986-992	1
Marucci, G., et al. (2015). "Pathological spectrum in recurrences of glioblastoma multiforme." Pathologica 107(1): 1-8	1
Minniti, G., et al. (2015). "Standard (60 Gy) or short-course (40 Gy) irradiation plus concomitant and adjuvant temozolomide for elderly patients with glioblastoma: a propensity-matched analysis." International journal of radiation oncology, biology, physics 91(1): 109-115	1
Mizumoto, M., et al. (2015). "Hyperfractionated concomitant boost proton radiotherapy for supratentorial glioblastoma multiforme." Radiotherapy and Oncology 115(SUPPL. 1): S400	1
Nct (2015). "Bioequivalence Study of Temozolomide in Patients With Primary Tumors of the Central Nervous System." A Randomized, Open Label, Two-way Crossover, Single Dose Bioequivalence Study Comparing Dralitem(R) Capsules to the Reference Drug Temodal(R) Capsules in Patients With Primary Tumors of the Central Nervous System Under Fasting Conditions.Prospective	3
Nguyen, A., et al. (2015). "An Innovative Fluorescent Semi-quantitative Methylation-specific PCR Method for the Determination of MGMT Promoter Methylation is Reflecting Intra-tumor Heterogeneity." Current cancer drug targets 15(7): 624-640	1
Peters, K., et al. (2015). "Low-dose naltrexone in newly diagnosed high-grade glioma: Placebo-controlled, double-blind, randomized pilot study." Neuro-Oncology 17(SUPPL. 5): v192	1
Rodon, J., et al. (2015). "First-in-human dose study of the novel transforming growth factor-beta receptor I kinase inhibitor LY2157299 monohydrate in patients with advanced cancer and glioma." Clinical Cancer Research 21(3): 553-560	1
Slat, E., et al. (2015). "Cell intrinsic circadaian regulation of temozolomide cytotoxicity in a glioblastoma model." Neuro-Oncology 17(SUPPL. 5): v127	3
Sneed, P. K., et al. (2015). "Adverse radiation effect after stereotactic radiosurgery for brain metastases: Incidence, time course, and risk factors." Journal of Neurosurgery 123(2): 373-386	1
Tabouret, E., et al. (2015). "Recurrence of glioblastoma after radio-chemotherapy is associated with an angiogenic switch to the CXCL12-CXCR4 pathway." Oncotarget 6(13): 11664-11675	1
Thomas, A. A., et al. (2015). "Dynamic contrast enhanced T1 MRI perfusion differentiates pseudoprogression from recurrent glioblastoma." Journal of neuro-oncology 125(1): 183-190	1

Umeda, K., et al. (2015). "Long-term efficacy of bevacizumab and irinotecan in recurrent pediatric glioblastoma." Pediatrics international : official journal of the Japan Pediatric Society 57(1): 169-171	1
Verreault, M., et al. (2015). "Determination of an optimal dosing schedule for combining Irinophore C TM and temozolomide in an orthotopic model of glioblastoma." Journal of controlled release : official journal of the Controlled Release Society 220(Pt A): 348-357	1
Wang, Z., et al. (2015). "Targeting miR-381-NEFL axis sensitizes glioblastoma cells to temozolomide by regulating stemness factors and multidrug resistance factors." Oncotarget 6(5): 3147-3164	1
Alonso-Basanta, M., et al. (2014). "A phase I study of nelfinavir concurrent with temozolomide and radiotherapy in patients with glioblastoma multiforme." Journal of neuro-oncology 116(2): 365-372	1
Barbagallo, G. M. V., et al. (2014). "Long-term therapy with temozolomide is a feasible option for newly diagnosed glioblastoma: a single-institution experience with as many as 101 temozolomide cycles." Neurosurgical focus 37(6): E4	1
Diebold, G., et al. (2014). "Management of glioblastoma: comparison of clinical practices and cost-effectiveness in two cohorts of patients (2008 versus 2004) diagnosed in a French university hospital." Journal of clinical pharmacy and therapeutics 39(6): 642-648	1
Fan, T.-Y., et al. (2014). "Inhibition of EZH2 reverses chemotherapeutic drug TMZ chemosensitivity in glioblastoma." International journal of clinical and experimental pathology 7(10): 6662-6670	1
Fortin, D., et al. (2014). "Intra-arterial carboplatin as a salvage strategy in the treatment of recurrent glioblastoma multiforme." Journal of neuro-oncology 119(2): 397-403	1
Han, S. J., et al. (2014). "Phase II trial of 7 days on/7 days off temozolmide for recurrent high-grade glioma." Neuro-Oncology 16(9): 1255-1262	1
Li, R., et al. (2014). "The temozolomide derivative 2T-P400 inhibits glioma growth via administration route of intravenous injection." Journal of neuro-oncology 116(1): 25-30	1
Marrero, L., et al. (2014). "Therapeutic efficacy of aldoxorubicin in an intracranial xenograft mouse model of human glioblastoma." Neoplasia (New York, N.Y.) 16(10): 874-882	1
McNamara, M. G., et al. (2014). "Conditional probability of survival and post-progression survival in patients with glioblastoma in the temozolomide treatment era." Journal of neuro-oncology 117(1): 153-160	1
McNamara, M. G., et al. (2014). "Factors impacting survival following second surgery in patients with glioblastoma in the temozolomide treatment era, incorporating neutrophil/lymphocyte ratio and time to first progression." Journal of neuro-oncology 117(1): 147-152	1
Muruganandham, M., et al. (2014). "3-Dimensional magnetic resonance spectroscopic imaging at 3 Tesla for early response assessment of glioblastoma patients during external beam radiation therapy." International journal of radiation oncology, biology, physics 90(1): 181-189	1
Porta, E., et al. (2014). "A neuroendocrine approach in the treatment of brain glioblastoma." Anticancer Research 34(10): 6118	1
Signore, M., et al. (2014). "Combined PDK1 and CHK1 inhibition is required to kill glioblastoma stem-like cells in vitro and in vivo." Cell death & disease 5: e1223	1
Soffietti, R., et al. (2014). "Bevacizumab and fotemustine for recurrent glioblastoma: a phase II study of AINO (Italian Association of Neuro-Oncology)." Journal of neuro-oncology 116(3): 533-541	1
van Vugt, V. A., et al. (2014). "Retrospective analysis of safety and feasibility of a 3 days on/11 days off temozolomide dosing regimen in recurrent adult malignant gliomas." CNS oncology 3(4): 257-265	1
Vannini, E., et al. (2014). "The bacterial protein toxin, cytotoxic necrotizing factor 1 (CNF1) provides long-term survival in a murine glioma model." BMC cancer 14: 449	1
Bailey, S., et al. (2013). "Diffuse intrinsic pontine glioma treated with prolonged temozolomide and radiotherapy--results of a United Kingdom phase II trial (CNS 2007 04)." European journal of cancer (Oxford, England : 1990) 49(18): 3856-3862	1
Bilal, M., et al. (2013). "Donor-derived metastatic melanoma in a liver transplant recipient established by DNA fingerprinting." Experimental and clinical transplantation : official journal of the Middle East Society for Organ Transplantation 11(5): 458-463	1
Dubrow, R., et al. (2013). "Time trends in glioblastoma multiforme survival: the role of temozolomide." Neuro-Oncology 15(12): 1750-1761	1

Filippi-Chiela, E. C., et al. (2013). "Resveratrol abrogates the temozolomide-induced G2 arrest leading to mitotic catastrophe and reinforces the temozolomide-induced senescence in glioma cells." BMC cancer 13: 147	1
Gielen, P. R., et al. (2013). "Connexin43 confers Temozolomide resistance in human glioma cells by modulating the mitochondrial apoptosis pathway." Neuropharmacology 75: 539-548	1
Graus, F., et al. (2013). "Patterns of care and outcome for patients with glioblastoma diagnosed during 2008-2010 in Spain." Neuro-Oncology 15(6): 797-805	1
Harrabi, S., et al. (2013). "Temozolomide in combination with carbon ion or photon irradiation in glioblastoma multiforme cell lines-does scheduling matter?" International Journal of Radiation Biology 89(9): 692-697	1
Ito, M., et al. (2013). "Early Chk1 phosphorylation is driven by temozolomide-induced, DNA double strand break- and mismatch repair-independent DNA damage." PloS one 8(5): e62351	1
Kaley, T. J., et al. (2013). "Phase I trial of temsirolimus (TEM) and perifosine (PER) for recurrent or progressive malignant glioma (MG)." Journal of Clinical Oncology 31(15 SUPPL. 1)	1
Keir, S. T., et al. (2013). "Mibefradil, a novel therapy for glioblastoma multiforme: cell cycle synchronization and interlaced therapy in a murine model." Journal of neuro-oncology 111(2): 97-102	3
Messali, A., et al. (2013). "The cost-effectiveness of temozolomide in the adjuvant treatment of newly diagnosed glioblastoma in the United States." Neuro-Oncology 15(11): 1532-1542	3
Passos, J., et al. (2013). "Narcolepsy secondary to a third ventricle glioblastoma." Journal of the Neurological Sciences 333(SUPPL. 1): e704	1
Pizer, B., et al. (2013). "Prolonged survival associated with the use of intraoperative carmustine (Gliadel) in a paediatric patient with recurrent grade III astrocytoma." British journal of neurosurgery 27(4): 516-518	1
Strowd, R. E., et al. (2013). "Impact of temozolomide on gonadal function in patients with primary malignant brain tumors." Journal of oncology pharmacy practice : official publication of the International Society of Oncology Pharmacy Practitioners 19(4): 321-327	1
Tobias, A. L., et al. (2013). "The timing of neural stem cell-based virotherapy is critical for optimal therapeutic efficacy when applied with radiation and chemotherapy for the treatment of glioblastoma." Stem cells translational medicine 2(9): 655-666	1
Wang, Q., et al. (2013). "Knockdown of RLIP76 expression by RNA interference inhibits invasion, induces cell cycle arrest, and increases chemosensitivity to the anticancer drug temozolomide in glioma cells." Journal of neuro-oncology 112(1): 73-82	1
Yeo, K. K., et al. (2013). "Durable response of intracranial cellular hemangioma to bevacizumab and temozolomide." Journal of neurosurgery. Pediatrics 11(6): 682-686	1
Zustovich, F., et al. (2013). "Sorafenib plus daily low-dose temozolomide for relapsed glioblastoma: a phase II study." Anticancer Research 33(8): 3487-3494	1
Aoki, Y., et al. (2012). "An experimental xenograft mouse model of diffuse pontine glioma designed for therapeutic testing." Journal of neuro-oncology 108(1): 29-35	1
Borges, K. S., et al. (2012). "Inhibition of Aurora kinases enhances chemosensitivity to temozolomide and causes radiosensitization in glioblastoma cells." Journal of Cancer Research and Clinical Oncology 138(3): 405-414	1
Demirci, U., et al. (2012). "Long term experience in high grade glial tumors with temozolomide." Journal of B.U.ON. : official journal of the Balkan Union of Oncology 17(2): 357-362	1
Grossman, R., et al. (2012). "The impact of bevacizumab on temozolomide concentrations in intracranial U87 gliomas." Cancer chemotherapy and pharmacology 70(1): 129-139	1
Kawanabe, Y., et al. (2012). "Radiation-induced spinal cord anaplastic astrocytoma subsequent to radiotherapy for testicular seminoma." Neurologia medico-chirurgica 52(9): 675-678	1
Koshy, M., et al. (2012). "Improved survival time trends for glioblastoma using the SEER 17 population-based registries." Journal of neuro-oncology 107(1): 207-212	1
Lawrence, Y. R., et al. (2012). "Improving prognosis of glioblastoma in the 21st century: who has benefited most?" Cancer 118(17): 4228-4234	3

Miller, R. J. H., et al. (2012). "Chemotherapy-associated steatohepatitis with temozolomide and dexamethasone." The Canadian journal of neurological sciences. Le journal canadien des sciences neurologiques 39(4): 547-549.	1
Noel, G., et al. (2012). "Waiting times before initiation of radiotherapy might not affect outcomes for patients with glioblastoma: a French retrospective analysis of patients treated in the era of concomitant temozolomide and radiotherapy." Journal of neuro-oncology 109(1): 167-175	1
Paccapelo, A., et al. (2012). "A retrospective pooled analysis of response patterns and risk factors in recurrent malignant glioma patients receiving a nitrosourea-based chemotherapy." Journal of translational medicine 10: 90	1
Pereira, A. M. and N. R. Biermasz (2012). "Treatment of nonfunctioning pituitary adenomas: what were the contributions of the last 10 years? A critical view." Annales d'endocrinologie 73(2): 111-116	3
Strik, H. M., et al. (2012). "Temozolomide dosing regimens for glioma patients." Current neurology and neuroscience reports 12(3): 286-293	3
Teres, S., et al. (2012). "2-Hydroxyoleate, a nontoxic membrane binding anticancer drug, induces glioma cell differentiation and autophagy." Proceedings of the National Academy of Sciences of the United States of America 109(22): 8489-8494	1
Addeo, R., et al. (2011). "A new schedule of fotemustine in temozolomide-pretreated patients with relapsing glioblastoma." Journal of neuro-oncology 102(3): 417-424	1
Chamberlain, M. C. (2011). "Radiographic patterns of relapse in glioblastoma." Journal of neuro-oncology 101(2): 319-323	1
Chaponis, D., et al. (2011). "Lonafarnib (SCH66336) improves the activity of temozolomide and radiation for orthotopic malignant gliomas." Journal of neuro-oncology 104(1): 179-189	1
Devito, N., et al. (2011). "Retrospective study of patients with brain metastases from melanoma receiving concurrent whole-brain radiation and temozolomide." Anticancer Research 31(12): 4537-4543	1
Freyschlag, C. F., et al. (2011). "Prolonged administration of temozolomide in adult patients with anaplastic glioma." Anticancer Research 31(11): 3873-3877	1
Grossman, S. A., et al. (2011). "Immunosuppression in patients with high-grade gliomas treated with radiation and temozolomide." Clinical cancer research : an official journal of the American Association for Cancer Research 17(16): 5473-5480	1
Henriksson, R., et al. (2011). "Impact of therapy on quality of life, neurocognitive function and their correlates in glioblastoma multiforme: a review." Journal of neuro-oncology 104(3): 639-646	3
Kim, Y.-H., et al. (2011). "Temozolomide during and after radiation therapy for WHO grade III gliomas: preliminary report of a prospective multicenter study." Journal of neuro-oncology 103(3): 503-512	1
Komatsu, F., et al. (2011). "Radiation-induced astrocytoma with rapid malignant transformation: case report." Neurologia medico-chirurgica 51(3): 243-246	1
Lawrence, Y. R., et al. (2011). "Delayed initiation of radiotherapy for glioblastoma: how important is it to push to the front (or the back) of the line?" Journal of neuro-oncology 105(1): 1-7	3
Lawrence, Y. R., et al. (2011). "Early toxicity predicts long-term survival in high-grade glioma." British journal of cancer 104(9): 1365-1371	1
Mhanna, H., et al. (2011). "Desensitization to Temozolomide." Journal of Allergy and Clinical Immunology 127(2 SUPPL. 1): AB197	3
Nct (2011). "Low Dose Naltrexone for Glioma Patients." Effects of Low Dose Naltrexone on Quality of Life in High Grade Glioma Patients: a Placebo-Controlled, Double-Blind Randomized Trial	2
Niewald, M., et al. (2011). "Toxicity after radiochemotherapy for glioblastoma using temozolomide--a retrospective evaluation." Radiation oncology (London, England) 6: 141	1
Parekh, C., et al. (2011). "Treatment of children with recurrent high grade gliomas with a bevacizumab containing regimen." Journal of neuro-oncology 103(3): 673-680	1
Rusthoven, K. E., et al. (2011). "Favorable prognosis in patients with high-grade glioma with radiation necrosis: the University of Colorado reoperation series." International journal of radiation oncology, biology, physics 81(1): 211-217	1
Takahashi, K., et al. (2011). "Case of atypical teratoid/rhabdoid tumor in an adult, with long survival." Brain tumor pathology 28(1): 71-76	1

van Nifterik, K. A., et al. (2011). "Anti-tumour effects by a trimodal combination of temozolomide, meloxicam and X-rays in cultures of human glioma cells." International Journal of Radiation Biology 87(2): 192-201	1
Walbert T, G. M. R. G. M. D. P. V. K. A. Y. W. K. C. C. A. B. G. C. C. H. H. S. (2011). "Combination of 6-thioguanine, capecitabine, and celecoxib with temozolomide or lomustine for recurrent high-grade glioma." Journal of neuro-oncology 102(2): 273	1
Aoki, T., et al. (2010). "Phase II study of ifosfamide, carboplatin, and etoposide in patients with a first recurrence of glioblastoma multiforme." Journal of Neurosurgery 112(1): 50-56	1
Barazzuol, L., et al. (2010). "A mathematical model of brain tumour response to radiotherapy and chemotherapy considering radiobiological aspects." Journal of theoretical biology 262(3): 553-565	1
Bauchet, L., et al. (2010). "Oncological patterns of care and outcome for 952 patients with newly diagnosed glioblastoma in 2004." Neuro-Oncology 12(7): 725-735	1
Brada, M., et al. (2010). "Temozolomide versus procarbazine, lomustine, and vincristine in recurrent high-grade glioma." Journal of clinical oncology : official journal of the American Society of Clinical Oncology 28(30): 4601-4608	1
Brandes, A. A., et al. (2010). "EORTC study 26041-22041: phase I/II study on concomitant and adjuvant temozolomide (TMZ) and radiotherapy (RT) with PTK787/ZK222584 (PTK/ZK) in newly diagnosed glioblastoma." European journal of cancer (Oxford, England : 1990) 46(2): 348-354	1
Cruickshanks, N., et al. (2010). "Can hsp90alpha-targeted siRNA combined with TMZ be a future therapy for glioma?" Cancer investigation 28(6): 608-614	1
Diez, B. D., et al. (2010). "Evaluation of the exposure equivalence of oral versus intravenous temozolomide." Cancer chemotherapy and pharmacology 65(4): 727-734	1
Harrison, C. (2010). "Patent watch." Nature reviews. Drug discovery 9(3): 184-185.	3
Hashizume, R., et al. (2010). "A human brainstem glioma xenograft model enabled for bioluminescence imaging." Journal of neuro-oncology 96(2): 151-159	1
Jahraus, C. D. and A. H. Friedman (2010). "Chemopotentiation by ultrafractionated radiotherapy in glioblastoma resistant to conventional therapy." Tumori 96(5): 771-775	1
Jung, T.-Y., et al. (2010). "Changes of the O6-methylguanine-DNA methyltransferase promoter methylation and MGMT protein expression after adjuvant treatment in glioblastoma." Oncology reports 23(5): 1269-1276	1
Karayan-Tapon, L., et al. (2010). "Prognostic value of O6-methylguanine-DNA methyltransferase status in glioblastoma patients, assessed by five different methods." Journal of neuro-oncology 97(3): 311-322	1
Lamoral-Theys, D., et al. (2010). "Long-term temozolomide treatment induces marked amino metabolism modifications and an increase in TMZ sensitivity in Hs683 oligodendroglioma cells." Neoplasia (New York, N.Y.) 12(1): 69-79	1
Le Calve, B., et al. (2010). "Long-term in vitro treatment of human glioblastoma cells with temozolomide increases resistance in vivo through up-regulation of GLUT transporter and aldo-keto reductase enzyme AKR1C expression." Neoplasia (New York, N.Y.) 12(9): 727-739	1
Marantidou, A., et al. (2010). "Steroid requirements during radiotherapy for malignant gliomas." Journal of neuro-oncology 100(1): 89-94	1
Milano, M. T., et al. (2010). "Patterns and timing of recurrence after temozolomide-based chemoradiation for glioblastoma." International journal of radiation oncology, biology, physics 78(4): 1147-1155	1
Mizumoto, M., et al. (2010). "Phase I/II Trial of Hyperfractionated Concomitant Boost Proton Radiotherapy for Supratentorial Glioblastoma Multiforme." International Journal of Radiation Oncology Biology Physics 77(1): 98-105	1
New, P. Z. and J. Jac (2010). "Phase II trial of chemotherapy with temozolomide in combination with topotecan for central nervous system (CNS) metastases of solid tumors." Journal of Clinical Oncology 28(15 SUPPL. 1)	1
Perry, J. R., et al. (2010). "Phase II trial of continuous dose-intense temozolomide in recurrent malignant glioma: RESCUE study." Journal of clinical oncology : official journal of the American Society of Clinical Oncology 28(12): 2051-2057	1

Platta, C. S., et al. (2010). "Current treatment strategies for brain metastasis and complications from therapeutic techniques: a review of current literature." American journal of clinical oncology 33(4): 398-407	3
Roberge, D., et al. (2010). "Temozolomide in combination with stereotactic radiosurgery for recurrent brain metastases: A phase I study." Journal of Clinical Oncology 28(15 SUPPL. 1)	1
Sampson, J. H., et al. (2010). "Immunologic escape after prolonged progression-free survival with epidermal growth factor receptor variant III peptide vaccination in patients with newly diagnosed glioblastoma." Journal of clinical oncology : official journal of the American Society of Clinical Oncology 28(31): 4722-4729	1
Sonoda, Y., et al. (2010). "O(6)-Methylguanine DNA methyltransferase determined by promoter hypermethylation and immunohistochemical expression is correlated with progression-free survival in patients with glioblastoma." International journal of clinical oncology 15(4): 352-358	1
Tsien, C., et al. (2010). "Parametric response map as an imaging biomarker to distinguish progression from pseudoprogression in high-grade glioma." Journal of clinical oncology : official journal of the American Society of Clinical Oncology 28(13): 2293-2299	1
van Genugten, J. A. B., et al. (2010). "Effectiveness of temozolomide for primary glioblastoma multiforme in routine clinical practice." Journal of neuro-oncology 96(2): 249-257	1
Watt, H. L., et al. (2010). "Receptor activation and inhibition in cellular response to chemotherapeutic combinational mimicries: the concept of divergent targeting." Journal of neuro-oncology 100(3): 345-361	1
Wen, P. Y. (2010). "Therapy for recurrent high-grade gliomas: does continuous dose-intense temozolomide have a role?" Journal of clinical oncology : official journal of the American Society of Clinical Oncology 28(12): 1977-1979.	4
Yaneva, M. P., et al. (2010). "Postoperative chemo-radiotherapy with temodal in patients with glioblastoma multiforme--survival rates and prognostic factors." Folia medica 52(1): 26-33	1
Bertagna, X., et al. (2009). "Cushing's disease." Best Practice and Research: Clinical Endocrinology and Metabolism 23(5): 607-623	3
Chamberlain, M. C. and J. Raizer (2009). "Extended exposure to alkylator chemotherapy: delayed appearance of myelodysplasia." Journal of neuro-oncology 93(2): 229-232	1
Combs, S. E. (2009). "Radiation therapy." Recent results in cancer research. Fortschritte der Krebsforschung. Progres dans les recherches sur le cancer 171: 125-140	3
DeAngelis, L. M. (2009). "Anaplastic glioma: how to prognosticate outcome and choose a treatment strategy. [corrected]." Journal of clinical oncology : official journal of the American Society of Clinical Oncology 27(35): 5861-5862.	3
Green, R. M., et al. (2009). "Bevacizumab for recurrent ependymoma." Neurology 73(20): 1677-1680	1
Grossman, S. A., et al. (2009). "Talampanel with standard radiation and temozolomide in patients with newly diagnosed glioblastoma: a multicenter phase II trial." Journal of clinical oncology : official journal of the American Society of Clinical Oncology 27(25): 4155-4161	1
Roldan, G. B., et al. (2009). "Population-based study of pseudoprogression after chemoradiotherapy in GBM." The Canadian journal of neurological sciences. Le journal canadien des sciences neurologiques 36(5): 617-622	1
Sato, Y., et al. (2009). "Diversity of DNA damage response of astrocytes and glioblastoma cell lines with various p53 status to treatment with etoposide and temozolomide." Cancer biology & therapy 8(5): 452-457	1
van den Bent, M. J., et al. (2009). "MGMT promoter methylation is prognostic but not predictive for outcome to adjuvant PCV chemotherapy in anaplastic oligodendroglial tumors: a report from EORTC Brain Tumor Group Study 26951." Journal of clinical oncology : official journal of the American Society of Clinical Oncology 27(35): 5881-5886	1
Virrey, J. J., et al. (2009). "Glioma-associated endothelial cells are chemoresistant to temozolomide." Journal of neuro-oncology 95(1): 13-22	1
Wick, W., et al. (2009). "NOA-04 randomized phase III trial of sequential radiochemotherapy of anaplastic glioma with procarbazine, lomustine, and vincristine or temozolomide." Journal of clinical oncology : official journal of the American Society of Clinical Oncology 27(35): 5874-5880	1

Widhalm, G., et al. (2009). "O(6)-methylguanine DNA methyltransferase immunoexpression in nonfunctioning pituitary adenomas: are progressive tumors potential candidates for temozolomide treatment?" Cancer 115(5): 1070-1080	1
Wyss, M., et al. (2009). "Early metabolic responses in temozolomide treated low-grade glioma patients." Journal of neuro-oncology 95(1): 87-93	1
Fathi, A.-R., et al. (2008). "Astroblastoma with rhabdoid features and favorable long-term outcome: report of a case with a 12-year follow-up." Pathology, research and practice 204(5): 345-351	1
Jalali, R. (2008). "Radiochemotherapy in patients with primary glioblastoma comparing two temozolomide dose regimens: is the survival really so impressive?: in regard to Combs et al. (Int J Radiat Oncol Biol Phys 2008;71:999-1005)." International journal of radiation oncology, biology, physics 72(5): 1622-1623.	3
Sabel, M. and A. Giese (2008). "Safety profile of carmustine wafers in malignant glioma: a review of controlled trials and a decade of clinical experience." Current medical research and opinion 24(11): 3239-3257	1
Trog, D., et al. (2008). "Up-regulation of vimentin expression in low-density malignant glioma cells as immediate and late effects under irradiation and temozolomide treatment." Amino acids 34(4): 539-545	1
Tuna-Malak, A. and A. Diramali (2008). "Radiotherapy-related tiredness in patients with glioblastoma multiforme (GBM)." Asian Pacific journal of cancer prevention : APJCP 9(3): 497-500	1
Ziobro, M., et al. (2008). "Effects of palliative treatment with temozolomide in patients with high-grade gliomas." Neurologia i neurochirurgia polska 42(3): 210-215	1
Chen, C. C., et al. (2007). "The Fanconi anemia (FA) pathway confers glioma resistance to DNA alkylating agents." Journal of molecular medicine (Berlin, Germany) 85(5): 497-509	1
Groves, M. D., et al. (2007). "A North American brain tumor consortium (NABTC 99-04) phase II trial of temozolomide plus thalidomide for recurrent glioblastoma multiforme." Journal of neuro-oncology 81(3): 271-277	1
Gururangan, S., et al. (2007). "Temozolomide in children with progressive low-grade glioma." Neuro-Oncology 9(2): 161-168	1
Powathil, G., et al. (2007). "Mathematical modeling of brain tumors: effects of radiotherapy and chemotherapy." Physics in medicine and biology 52(11): 3291-3306	1
Yamini, B., et al. (2007). "Inhibition of nuclear factor-kappaB activity by temozolomide involves O6-methylguanine induced inhibition of p65 DNA binding." Cancer research 67(14): 6889-6898	1
Yamini, B., et al. (2007). "Adenovirally delivered tumor necrosis factor-alpha improves the antiglioma efficacy of concomitant radiation and temozolomide therapy." Clinical cancer research : an official journal of the American Association for Cancer Research 13(20): 6217-6223	1
Laber, D. A., et al. (2006). "A phase II study of extended dose temozolomide and thalidomide in previously treated patients with metastatic melanoma." Journal of Cancer Research and Clinical Oncology 132(9): 611-616	1
Rehman, S., et al. (2006). "A case report of a recurrent intracranial ependymoma treated with temozolomide in remission 10 years after completing chemotherapy." American journal of clinical oncology 29(1): 106-107.	1
Tafuto, S., et al. (2006). "Phase I study of temozolomide and lomustine in the treatment of high grade malignant glioma." Frontiers in bioscience : a journal and virtual library 11: 502-505	1
Bobola, M. S., et al. (2005). "Apurinic/apyrimidinic endonuclease activity is associated with response to radiation and chemotherapy in medulloblastoma and primitive neuroectodermal tumors." Clinical cancer research : an official journal of the American Association for Cancer Research 11(20): 7405-7414	1
Bobola, M. S., et al. (2005). "O6-methylguanine-DNA methyltransferase, O6-benzylguanine, and resistance to clinical alkylators in pediatric primary brain tumor cell lines." Clinical cancer research : an official journal of the American Association for Cancer Research 11(7): 2747-2755	1
Boiardi, A., et al. (2005). "Systemic temozolomide combined with loco-regional mitoxantrone in treating recurrent glioblastoma." Journal of neuro-oncology 75(2): 215-220	1

Cohen, M. H., et al. (2005). "Food and Drug Administration Drug approval summary: temozolomide plus radiation therapy for the treatment of newly diagnosed glioblastoma multiforme." Clinical cancer research : an official journal of the American Association for Cancer Research 11(19 Pt 1): 6767-6771	3
Combs, S. E., et al. (2005). "Temozolomide combined with irradiation as postoperative treatment of primary glioblastoma multiforme. Phase I/II study." Strahlentherapie und Onkologie : Organ der Deutschen Rontgengesellschaft ... [et al] 181(6): 372-377	1
Khatami, R., et al. (2005). "Hypersomnia in an epilepsy patient treated with levetiracetam." Epilepsia 46(4): 588-589	1
Rutz, H. P., et al. (2005). "Avoiding glucocorticoid administration in a neurooncological case." Cancer biology & therapy 4(11): 1186-1189	1
Tentori, L., et al. (2005). "Brain distribution and efficacy as chemosensitizer of an oral formulation of PARP-1 inhibitor GPI 15427 in experimental models of CNS tumors." International journal of oncology 26(2): 415-422	1
Weller, M., et al. (2005). "Temozolomide: a milestone in the pharmacotherapy of brain tumors." Future oncology (London, England) 1(6): 747-754	3
Wemmert, S., et al. (2005). "Patients with high-grade gliomas harboring deletions of chromosomes 9p and 10q benefit from temozolomide treatment." Neoplasia (New York, N.Y.) 7(10): 883-893	1
Bafaloukos, D., et al. (2004). "The effect of temozolomide-based chemotherapy in patients with cerebral metastases from melanoma." Melanoma research 14(4): 289-294	1
Hegi, M. E., et al. (2004). "Clinical trial substantiates the predictive value of O-6-methylguanine-DNA methyltransferase promoter methylation in glioblastoma patients treated with temozolomide." Clinical cancer research : an official journal of the American Association for Cancer Research 10(6): 1871-1874	1
Kortmann, R.-D., et al. (2004). "Immediate postoperative radiotherapy or "watch and wait" in the management of adult low-grade glioma?" Strahlentherapie und Onkologie : Organ der Deutschen Rontgengesellschaft ... [et al] 180(7): 408-418	1
Balana, C., et al. (2003). "O6-methyl-guanine-DNA methyltransferase methylation in serum and tumor DNA predicts response to 1,3-bis(2-chloroethyl)-1-nitrosourea but not to temozolamide plus cisplatin in glioblastoma multiforme." Clinical cancer research : an official journal of the American Association for Cancer Research 9(4): 1461-1468	1
Hirose, Y., et al. (2003). "Delayed repletion of O6-methylguanine-DNA methyltransferase resulting in failure to protect the human glioblastoma cell line SF767 from temozolomide-induced cytotoxicity." Journal of Neurosurgery 98(3): 591-598	1
Kortmann, R.-D., et al. (2003). "Radiochemotherapy of malignant glioma in adults. Clinical experiences." Strahlentherapie und Onkologie : Organ der Deutschen Rontgengesellschaft ... [et al] 179(4): 219-232	3
Miknyoczki, S. J., et al. (2003). "Chemopotentiation of temozolomide, irinotecan, and cisplatin activity by CEP-6800, a poly(ADP-ribose) polymerase inhibitor." Molecular cancer therapeutics 2(4): 371-382	1
Bocangel, D. B., et al. (2002). "Multifaceted resistance of gliomas to temozolomide." Clinical cancer research : an official journal of the American Association for Cancer Research 8(8): 2725-2734	3
Cher, L. M. (2001). "10: Cancer and the nervous system." Medical Journal of Australia 175(5): 277-282	3
Haney, S. M., et al. (2001). "Mapping therapeutic response in a patient with malignant glioma." Journal of computer assisted tomography 25(4): 529-536	1
Rao, R. D., et al. (2001). "Late recurrence of a primitive neuro-ectodermal tumor." Oncology 61(3): 189-191	1
van den Bent, M. J., et al. (2001). "Temozolomide chemotherapy in recurrent oligodendroglioma." Neurology 57(2): 340-342	1
Heimberger, A. B., et al. (2000). "Temozolomide delivered by intracerebral microinfusion is safe and efficacious against malignant gliomas in rats." Clinical cancer research : an official journal of the American Association for Cancer Research 6(10): 4148-4153	1
Patel, V. J., et al. (2000). "Schedule-dependent activity of temozolomide plus CPT-11 against a human central nervous system tumor-derived xenograft." Clinical cancer research : an official journal of the American Association for Cancer Research 6(10): 4154-4157	1

Yung, W. K., et al. (2000). "A phase II study of temozolomide vs. procarbazine in patients with glioblastoma multiforme at first relapse." British journal of cancer 83(5): 588-593	1
Wedge, S. R. and E. S. Newlands (1996). "O6-benzylguanine enhances the sensitivity of a glioma xenograft with low O6-alkylguanine-DNA alkyltransferase activity to temozolomide and BCNU." British journal of cancer 73(9): 1049-1052	1

Table S2

**Reference**	**Reason for Exclusion**
Anselmo, P., et al. (2021). "Clinical characterization of glioblastoma patients living longer than 2 years: A retrospective analysis of two Italian institutions." Asia-Pacific journal of clinical oncology 17(3): 273-279	1
Nct (2019). "Short Course Chemo-Radiation Therapy for Patients With Newly Diagnosed Glioblastoma." Short Course Chemo-Radiation Therapy for Patients With Newly Diagnosed Glioblastoma: comparison of Two Established Schedules	3
Ryu, J. Y., et al. (2019). "Effect of anti-epileptic drugs on the survival of patients with glioblastoma multiforme: A retrospective, single-center study." PloS one 14(12): e0225599	1
Vallathol, S. H. (2018). "A study of adherence to oral temozolomide chemotherapy in central nervous system tumors." Annals of Oncology 29(Supplement 9)	1
Nct (2016). "Temozolomide Chronotherapy for High Grade Glioma." A Randomized Feasibility Study Evaluating Temozolomide Chronotherapy for High Grade Glioma	3
Ctri (2014). "To monitor the safety and to assess the bioequivalence of Temozolomide 250 mg capsule of Sun Pharmaceutical Industries Ltd. and Temodal (Temozolomide) 250mg capsule of Merck Canada Inc. in cancer patients with high grade glioma, under fasting conditions." A RANDOMIZED, TWO TREATMENT, TWO PERIOD, TWO SEQUENCE, SINGLE DOSE, CROSSOVER, BIOEQUIVALENCE STUDY OF TEMOZOLOMIDE 250 MG CAPSULES OF SUN PHARMACEUTICAL INDUSTRIES LIMITED, INDIA AND TEMODAL (TEMOZOLOMIDE) 250 MG CAPSULES OF MERCK CANADA INC., IN PATIENTS WITH HIGH GRADE GLIOMA, UNDER FASTING CONDITIONS	3
Graus, F., et al. (2013). "Patterns of care and outcome for patients with glioblastoma diagnosed during 2008-2010 in Spain." Neuro-Oncology 15(6): 797-805	1
Erpolat, O. P., et al. (2009). "Outcome of newly diagnosed glioblastoma patients treated by radiotherapy plus concomitant and adjuvant temozolomide: a long-term analysis." Tumori 95(2): 191-197	1
Brandes, A. A., et al. (2008). "MGMT promoter methylation status can predict the incidence and outcome of pseudoprogression after concomitant radiochemotherapy in newly diagnosed glioblastoma patients." Journal of clinical oncology : official journal of the American Society of Clinical Oncology 26(13): 2192-2197	1
Minniti G, D. S. V. M. R. F. F. B. A. V. M. O. M. F. D. P. U. L. G. T. V. M. E. R. (2008). "Radiotherapy plus concomitant and adjuvant temozolomide for glioblastoma in elderly patients." Journal of neuro-oncology 88(1): 97	1
Taal, W., et al. (2008). "Incidence of early pseudo-progression in a cohort of malignant glioma patients treated with chemoirradiation with temozolomide." Cancer 113(2): 405-410	1
Caroli, M., et al. (2007). "Temozolomide in glioblastoma: results of administration at first relapse and in newly diagnosed cases. Is still proposable an alternative schedule to concomitant protocol?" Journal of neuro-oncology 84(1): 71-77	1
Parlato, C., et al. (2006). "Surgery, radiotherapy and temozolomide in treating high-grade gliomas." Frontiers in bioscience : a journal and virtual library 11: 1280-1283	3
Wong, S., et al. (2006). "Phase II study of two-weekly temozolomide in patients with high-grade gliomas." Journal of clinical neuroscience : official journal of the Neurosurgical Society of Australasia 13(1): 18-22	1

Wick, W., et al. (2004). "One week on/one week off: A novel active regimen of temozolomide for recurrent glioblastoma." Neurology 62(11): 2113-2115	1
Trippoli, S., et al. (2003). "Survival of patients with recurrent malignant glioma treated with temozolomide: a retrospective observational study." Drugs in R&D 4(5): 285-291	1
